# The effect of urinary incontinence status during pregnancy and delivery mode on incontinence postpartum. A cohort study*

**DOI:** 10.1111/j.1471-0528.2008.02107.x

**Published:** 2009-02-11

**Authors:** SL Wesnes, S Hunskaar, K Bo, G Rortveit

**Affiliations:** a Section for General Practice, Department of Public Health and Primary Health Care, University of BergenBergen, Norway; b Department of Sports Medicine, The Norwegian School of Sports SciencesOslo, Norway; c Research Unit for General Practice, Unifob HealthBergen, Norway

**Keywords:** Caesarean section, cohort study, postpartum, primiparity, urinary incontinence, vaginal birth

## Abstract

**Objective:**

The objectives of this study were to investigate prevalence of urinary incontinence at 6 months postpartum and to study how continence status during pregnancy and mode of delivery influence urinary incontinence at 6 months postpartum in primiparous women.

**Design:**

Cohort study.

**Setting:**

Pregnant women attending routine ultrasound examination were recruited to the Norwegian Mother and Child Cohort Study (MoBa).

**Population:**

A total of 12 679 primigravidas who were continent before pregnancy.

**Methods:**

Data are from MoBa, conducted by the Norwegian Institute of Public Health. Data are based on questionnaires answered at week 15 and 30 of pregnancy and 6 months postpartum.

**Main outcome measures:**

Urinary incontinence 6 months postpartum is presented as proportions, odds ratios and relative risks (RRs).

**Results:**

Urinary incontinence was reported by 31% of the women 6 months after delivery. Compared with women who were continent during pregnancy, incontinence was more prevalent 6 months after delivery among women who experienced incontinence during pregnancy (adjusted RR 2.3, 95% CI 2.2–2.4). Adjusted RR for incontinence after spontaneous vaginal delivery compared with elective caesarean section was 3.2 (95% CI 2.2–4.7) among women who were continent and 2.9 (95% CI 2.3–3.4) among women who were incontinent in pregnancy.

**Conclusion:**

Urinary incontinence was prevalent 6 months postpartum. The association between incontinence postpartum and mode of delivery was not substantially influenced by incontinence status in pregnancy. Prediction of a group with high risk of incontinence according to mode of delivery cannot be based on continence status in pregnancy.

## Introduction

Urinary incontinence is a common condition among women.^1^–^4^ Pregnancy and delivery seem to be major risk factors among young and middle-aged women.^4^,^5^ However, the reported prevalence of urinary incontinence varies widely both during^6^–^8^ and after pregnancy.^9^–^11^ Urinary incontinence postpartum is a disorder consisting of incontinence starting before, during and after pregnancy. The group has heterogeneous pathophysiology, and different risk factors may exist depending on the time of origin of the disorder. Urinary incontinence starting before or during pregnancy is likely to be associated with incontinence after pregnancy. Some studies have found it to be an independent risk factor for incontinence postpartum^12^–^14^ and later in life,^15^,^16^ but one study found no such association.^17^ The role of urinary incontinence during pregnancy, especially incident urinary incontinence, has so far received little attention as a potential risk factor for incontinence after pregnancy and later in life.^11^,^18^

A series of risk factors seems to be involved in urinary incontinence postpartum and later in life, among which there is growing evidence for the impact of delivery mode.^13^,^19^,^20^ Few authors have studied the effect of delivery mode among primiparous women. We could find only one such study that reported analyses stratified for continence status during pregnancy.^11^ Some studies dealing with these issues have methodological weaknesses like poor outcome measures, recall bias and retrospective design.^11^,^21^ In addition, there are problems with small study groups, small numbers of caesarean sections (CS), missing information on elective and non-elective CS and instrumental vaginal deliveries and no adjustments for important confounders like age and body mass index (BMI).^10^,^12^,^17^,^18^,^22^–^24^ We planned the present study to meet some of these challenges.

The Norwegian Mother and Child Cohort Study (MoBa) is a large population-based cohort of pregnant women, with several years of follow up, aiming at investigating health issues among both mothers and children.^25^ The study population in the present substudy consists of primigravid women who were continent before pregnancy, as this is the best available clinical model of a pelvis unexposed to known pregnancy-related risk factors,^26^ and thereby it is the best population to assess the risk of urinary incontinence associated with pregnancy and delivery. Our objective was first, to investigate the incidence and prevalence of urinary incontinence 6 months after delivery; second, to investigate the impact of continence status in week 30 of pregnancy on urinary incontinence 6 months postpartum and third, to study how mode of delivery may interact with continence status in pregnancy to increase or reduce the risk of urinary incontinence 6 months postpartum.

## Materials and methods

There are approximately 55 000 births in Norway annually. The MoBa invited approximately 29 000 pregnant women annually from 1999 to participate in the study, aiming at a study population of 100 000 women.^25^ A total of 39 of about 50 hospitals and maternity units in Norway with more than 100 births annually participate in the study. Two weeks before the routine pregnancy ultrasound examination, an invitation was mailed to the pregnant women. By 2006, 45% of the invited women had accepted to participate by informed written consent. MoBa is still recruiting in 2008. The women were asked only once. However, given participation, response in follow-up studies was strongly emphasised.

The study obtained data by postal questionnaires at six time points from week 15 in pregnancy to 7 years after birth. In this study, we used data set from questionnaire 1 (week 15 of pregnancy), questionnaire 3 (week 30 of pregnancy) and questionnaire 4 (6 months postpartum). We included women in their first pregnancy, singletons only, who reported having been continent before pregnancy. Questionnaire 4 was answered by 87% of the women who answered the questionnaire 3. Descriptive data based on questionnaires 1 and 3 have been published previously.^7^

We used a symptom-based questionnaire based on the terminology of the International Continence Society (ICS).^27^ The women were asked about current leakage. Incontinence was reported as occurring when coughing/laughing/sneezing, when running/jumping or if they had leakage accompanied by a strong urge to void. Frequency (never, one to four times per month, one to six times per week, once a day and more than once a day) and amount (droplets and larger volumes) were registered. The two last frequency groups were categorised into ‘Once or more a day’ for the analyses. We defined a case of urinary incontinence when the woman reported frequency of leakage or amount or both. Women who reported no incontinence but answered the frequency question were regarded incontinent (*n*= 110). Women who failed to answer the incontinence questions postpartum (*n*= 186) and women without information on continence status during pregnancy (*n*= 16) were included in analyses with missing values. We defined severe urinary incontinence as leaking ‘Larger amounts’ or ‘Once or more a day’ or both.

Women confirming loss of urine in association with coughing, laughing, sneezing, running or jumping were defined as having a stress incontinence component. Women with urgency accompanying loss of urine were defined as having an urge incontinence component. We use the term ‘stress urinary incontinence’ for women who had a stress component only, while ‘urge urinary incontinence’ denotes women who had an urge component only. Women who had symptoms of both components are referred to as having mixed urinary incontinence, according to standardised terminology of lower urinary tract symptoms.^27^

The standard data set from the Medical Birth Registry of Norway was included in the database for the MoBa. The Norwegian Data Inspectorate has approved the linkage of the databases. If the Medical Birth Registry did not have information on previous births, the women were defined as nulliparous and included in this study. The Medical Birth Registry holds information on mode of delivery. CS is categorised as ‘elective CS’, ‘acute caesarean section intended as elective CS’, ‘acute caesarean section intended as spontaneous vaginal delivery’ or ‘unspecified CS’ in the registry. We use the term ‘non-elective CS’ to denote the categories of acute CS intended as elective CS, acute CS intended as spontaneous vaginal delivery and unspecified CS as a group. Vaginal delivery is categorised as ‘spontaneous vaginal delivery’ (SVD), ‘forceps delivery’ or ‘vacuum delivery’. Continence status during pregnancy and mode of delivery were the exposures in this study.

Age was obtained in week 15 of pregnancy. Based on prevalence curves of urinary incontinence during pregnancy, we categorised age into four age groups (≤26, 27–30, 31–34 and ≥35 years). The height was reported at week 15. We excluded outliers by only including values from 140 cm. BMI was calculated as weight in kilograms/(height in metres)^2^. For BMI, we used the weight reported 6 months postpartum. Outliers for weight were excluded; values from 40–180 kg were included. BMI was categorised into four groups: <20 (underweight), 20–24.9 (normal weight), 25–29.9 (overweight) and ≥30 kg/m^2^ (obese).

The following potential confounders were explored: age, BMI, sex of baby, head circumference, baby’s weight, Apgar score (1 and 5 minutes), fetal presentation at delivery (normal occipital, breech, transverse, abnormal fetal head presentation and other), birth time (minutes), prolonged labour, perineal tear grade 3–4 and induction (amniotomy, oxytocin and prostaglandins). The Medical Birth Registry’s definitions of the variables are based on the Clinical Guidelines in Obstetrics.^28^ Age and BMI were identified as confounders in this material and are therefore the only variables included in adjusted analyses.

The Norwegian Data Inspectorate approved the MoBa study in 1996 and renewed the approval in 2003. The Regional Ethics Committee for Medical Research, Health Region II, has also endorsed the project.

We defined cumulative incidence of incontinence as any incontinence developed after delivery among women who were continent during pregnancy. Confounding was evaluated and adjusted for by multivariable logistic regression analyses and crosstabs analyses. Effect modification of continence status on the effect of SVD compared with elective CS was tested by use of interaction terms in multivariable logistic regression analyses. We treated independent variables as categorical. Odds ratios were the preliminary outcome measure in our analyses. All odds ratios and odds ratio confidence intervals (CI) were then converted to relative risks (RRs) and corresponding CI by use of the formula RR = OR/((1 −*P*) + (OR ×*P*)).^29^ In this formula, *P*is the prevalence of urinary incontinence in the unexposed group. Data are presented as mean, odds ratio and RR with 95% CI. *P*values less than 5% were considered statistically significant. SPSS 15.0 for Windows (SPSS Inc., Chicago, IL, USA) was used for statistical analyses.

## Results

A total of 12 679 primigravid women were included in this substudy. All women were continent before pregnancy. Mean age was 28 years (range 15–45 years), and mean BMI was 24.1 kg/m^2^ (range 14–54 kg/m^2^). Urinary incontinence was reported by 31% (3991/12 679) of women 6 months after delivery. A total of 14% (1815/12 679) of the women had delivered by CS. Descriptive data for mode of delivery and continence status during pregnancy are presented in Table 1. Women who delivered by CS had a higher age and BMI than those who delivered vaginally. More women who delivered by CS had babies with diverging fetal presentation and higher head circumference compared with women with vaginal delivery. Women having urinary incontinence during pregnancy had a higher age and BMI compared with those who were continent during pregnancy. Stress incontinence was the most common type of incontinence 6 months postpartum (*n*= 1728/12 679; 14%). Only 5% (186/3991) had urinary leakage ≥1 per day and 5% (212/3991) leaked larger amounts. A total of 43 women had urinary leakage ≥1 per day and simultaneously reported leaking larger amounts. The urinary frequency and amount of leakage were unaltered after delivery among the majority of women (data not shown).

**Table 1:** Descriptive baseline values of the exposure groups in primigravida women who were continent before pregnancy

	**Mode of delivery**				**Continence status in week 30**			
	**Caesarean section (*n*= 1815)**		**Vaginal delivery (*n*= 10 864)**		**Urinary continent during pregnancy (*n*= 7561)**		**Urinary incontinent during pregnancy (*n*= 5102)**	
**Characteristics of mother**								
Urinary incontinence during pregnancy	691	38%	4411	41%	NA	NA	5102	100%
Urinary continence during pregnancy	1120	62%	6441	59%	7561	100%	NA	NA
Age (years)*	28.9	4.3	27.9	4.5	27.9	4.2	28.2	4.4
BMI (kg/m^2^)*	25.4	4.7	23.9	4.1	23.9	4.1	24.4	4.4
**Characteristic of the neonatals**								
Gender (% boys)	1001	55%	5307	50%	3713	49%	2588	51%
Head circumference (cm)*	35.4	2.1	35.1	1.7	35.2	1.8	35.1	1.6
Weight (g)*	3500	782	3532	494	3528	546	3525	545
Apgar 1 minute*	8.4	1.6	8.6	1.2	8.6	1.3	8.6	1.2
Apgar 5 minutes*	9.2	1.1	9.4	0.8	9.3	0.9	9.4	0.8
**Mode of delivery**								
Elective CS	355	20%	NA	NA	227	3%	128	3%
Acute CS intended as elective CS	45	3%	NA	NA	31	0%	14	0%
Acute CS intended as SVD	1348	74%	NA	NA	824	1	521	10%
Unspecified CS	67	4%	NA	NA	38	1%	28	1%
SVD	NA	NA	8908	82%	5219	69%	3677	72%
Forceps	NA	NA	309	3%	189	2%	120	2%
Vacuum	NA	NA	1647	15%	1033	14%	614	12%
**Fetal presentation**								
Normal occipital	1160	64%	9928	94%	6609	90%	4466	89%
Breech	414	23%	204	2%	354	5%	263	5%
Transverse	7	0%	2	0%	5	0%	4	0%
Abnormal fetal head presentation	210	12%	390	4%	363	5%	235	5%
Other	23	1%	57	1%	50	1%	30	1%
Duration*								
Duration of birth (minutes)	1258	653	1206	687	1203	687	1231	673
**Rupture**								
Rupture grade 3–4	NA	NA	833	8%	483	6%	351	7%
**Induction**								
Amniotomy	87	5%	301	3%	227	3%	160	3%
Oxytocin	112	6%	387	4%	268	4%	230	5%
Prostaglandines	285	16%	795	7%	623	8%	456	9%
**Type of incontinence 6 months postpartum**								
Stress incontinence	112	6%	1616	15%	651	9%	1074	21%
Urge incontinence	90	5%	859	8%	471	6%	475	9%
Mixed incontinence	79	4%	1235	11%	440	6%	872	17%
**Severity of incontinence 6 months postpartum**								
Severe urinary incontinence	27	1%	331	3%	118	2%	239	5%

NA, not applicable.

Data are given by two exposure variables: mode of delivery and continence status in week 30. All data are given as number and proportion unless otherwise stated. Women with missing data were excluded in analyses of continence status in week 30.

* Data are presented as mean and SD.

### Impact of continence status during pregnancy on postpartum incontinence

Urinary incontinence 6 months postpartum according to continence status in week 30 of pregnancy is presented in Table 2. A total of 52% (2605/5026) of the women who were incontinent in pregnancy were continent 6 months postpartum. Urinary incontinence in week 30 of pregnancy was a statistically significant risk factor for persistent urinary incontinence postpartum, with an adjusted RR of 2.3 compared with women who were continent in week 30. A total of 21% (1562/7561) of the women, who were continent before and during pregnancy, had become incontinent 6 months postpartum (cumulative incidence). The strongest associated factors for de novo urinary incontinence in adjusted analysis were forceps delivery (RR 4.0, 95% CI 2.6–5.8), SVD (RR 3.2, 95% CI 2.1–4.7), vacuum delivery (RR 3.2, 95% CI 2.1–4.7), all compared with elective CS. Additionally, age >35 years (RR 1.8, 95% CI 1.5–2.1) and BMI >30 kg/m^2^ (RR 1.8, 95% CI 1.5–2.1) were significantly associated with de novo incontinence.

**Table 2:** Prevalence of incontinence 6 months after delivery among women who were continent before pregnancy according to mode of delivery and continence status in week 30

	**Urinary incontinence 6 months after delivery**						
	**All**	**Incontinent women**		**Unadjusted OR**	Adjusted OR*	Adjusted RR*	**95% CI**
	***N***	***n***	**%**				
**Continence status week 30**							
Continent	7451	1562	21	1	1	1	Reference
Incontinent	5026	2421	48	3.5	3.5	2.3	2.2–2.4
**Mode of delivery**							
Elective CS	354	43	13	1	1	1	Reference
Acute CS intended as elective CS	45	7	16	1.3	1.4	1.2	0.6–3.1
Acute CS intended as SVD	1322	220	17	1.4	1.5	1.3	1.0–2.1
Unspecified CS	66	11	17	1.4	1.5	1.3	0.7–3.2
SVD	8908	3010	34	3.7	4.7	3.2	2.5–3.9
Vacuum	1647	587	36	4.0	5.1	3.3	2.6–4.0
Forceps	309	113	37	4.2	5.5	3.5	2.6–4.3

*P*value ≤0.001 for all estimates except for acute CS intended as elective CS, acute CS intended as SVD and unspecified CS. Women with missing data were excluded in analyses of continence status in week 30.

* Adjusted for age and BMI.

### Impact of delivery mode

The prevalence of urinary incontinence 6 months postpartum was in general lower for the CS group (Table 2). There was no statistically significant increased risk associated with any of the three groups of non-elective CS compared with the elective CS group. When these three groups were analysed together, the difference was of borderline significance (RR 1.4, 95% CI 1.0–1.8). The adjusted RR for urinary incontinence postpartum among women having a SVD was 3.2 compared with elective CS. The incidences of urinary incontinence among women who were continent during pregnancy by the different types of delivery are shown in Table 3. After forceps delivery, 30% became incontinent.

**Table 3:** Number (*n*), percentage and adjusted odds ratio and RR for urinary incontinence 6 months postpartum by delivery mode, stratified for continence status during pregnancy

	**Continent during pregnancy**					**Incontinent during pregnancy**				
	***n***	**%**	**OR**	**RR**	**CI**	***n***	**%**	**OR**	**RR**	**CI**
Elective CS	18	8	1	1	Reference	25	20	1	1	Reference
Acute CS intended as elective CS	4	13	1.6	1.4	0.4–4.1	3	21	1.4	1.3	0.3–2.9
Acute CS intended as SVD	66	8	1.0	1.0	0.6–1.7	153	30	1.9	1.6	1.1–2.2
Unspecified CS	3	8	0.7	0.7	0.2–2.8	8	29	2.0	1.7	0.7–2.8
SVD	1166	23	3.9	3.2	2.1–4.7	1837	51	5.5	2.9	2.3–3.4
Vacuum	250	26	3.9	3.2	2.1–4.6	337	56	6.4	3.1	2.4–3.6
Forceps	55	30	5.5	4.0	2.6–5.8	58	50	4.9	2.8	2.0–3.4

### The combined impact of delivery mode and incontinence status during pregnancy

In the group of women who were continent during pregnancy, 8% of the women were incontinent after elective CS and 20% were incontinent after SVD, representing an absolute increase of 12%. The corresponding percentages for women, who were incontinent during pregnancy, were 23 and 51% with an absolute increase of 28% (Table 3). The percentages were approximately the same when comparing all CS to all vaginal deliveries. In adjusted analysis, the risk of incontinence 6 months after acute CS intended as SVD was statistically significant (RR 1.6) compared with elective CS among women who were incontinent during pregnancy (Table 3). When the three groups of non-elective CS were analysed together, the difference was still significant (RR 1.6, 95% CI 1.1–2.2). SVD was a strong and statistically significant risk factor for incontinence 6 months after delivery compared with elective CS both among women who were continent in week 30 of pregnancy (RR 3.2) and for women incontinent in week 30 (RR 2.9) (Table 3). The difference in RR between these groups was not statistically significant.

## Comment

In this large cohort of primigravid women who were continent before pregnancy, we found considerably raised risks for urinary incontinence postpartum among those who developed urinary incontinence during pregnancy compared with those who were continent. The effect of mode of delivery on urinary incontinence postpartum did not depend on continence status during pregnancy.

We found an odds ratio of 3.5 for urinary incontinence 6 months postpartum among women who were incontinent during pregnancy compared with those who were continent at that time. When reanalysing available data in previously published articles for comparison, odds ratios for urinary incontinence postpartum among primiparous women by continence status during pregnancy vary from 2.5 to 9.2.^9^,^11^,^12^,^24^,^30^–^33^ We have identified four studies investigating the relationship between continence status during pregnancy and continence status postpartum in previously continent primigravid women, showing odds ratios of 3.1,^12^ 4.3,^11^ 5.4^9^ and 7.8.^33^ Reasons for the higher odds ratios in three of these articles compared with our study might be higher age of the study population,^33^ restriction to stress urinary incontinence,^33^ investigations 3 months postpartum^9^,^11^ and the use of interviewers.^33^ Methodological issues like small study populations^9^,^33^ and retrospective design^11^,^12^ probably contribute to less precision in the results. In addition, there was no possibility for adjustments of odds ratio in our reanalyses. Many authors claim that urinary incontinence during pregnancy is an important predictor for urinary incontinence postpartum and later in life.^10^,^13^,^15^,^30^,^32^,^34^ Glazener *et al.*^11^ was the only group investigating primiparous women who were continent before pregnancy, stratified for continence status during pregnancy and then analysed delivery parameters, similar to our approach. For comparison, we set CS as reference group in Glazener’s study and any CS as reference group in our material. Reanalysed this way, the odds ratio for urinary incontinence after vaginal delivery among women who were continent during pregnancy was 3.6 in Glazener’s study and 3.3 in ours. Among women who were incontinent during pregnancy, the odds ratios were 2.6 and 2.6, respectively. Although Glazener *et al*. used a retrospective design with data collection 3 months postpartum, our results correspond very well with theirs.

Incidence of urinary incontinence postpartum among primiparous women who were continent both before and during pregnancy varies from 5 to 20%.^9^,^11^,^26^ We report a cumulative incidence 6 months postpartum of 21%. Reasons for this high incidence may be lower CS rates and higher rates of instrumental vaginal delivery in our study compared with the other studies.^9^,^11^,^26^ Also, we have used a low threshold to define urinary incontinence. Our cumulative incidences on urinary incontinence after CS, SVD and instrumental delivery were, however, equal to other studies.^26^ Even though we report high incidence and prevalence of urinary incontinence in this study, only a fraction of the women reported frequent leakage of urine or leaking larger amounts. Other studies have found that most pregnant women are not bothered by their urinary incontinence.^35^

MoBa invited annually 29 000 pregnant women in Norway to participate, underscoring that the target population of MoBa was a population-based and nonselected sample. The response rate among primigravid women was 45%. There may be many reasons for the initial low response rate,^36^ for instance resistance to commitment in a comprehensive study with questionnaires of 16 pages. The study population may thus not be representative for pregnant women in every aspect. There were, however, only minor differences between the MoBa participants and their deliveries compared with all births in Norway in the same period concerning distribution of demographic variables.^25^ There was a socio-economic gradient that influenced prevalence estimates, as women in lower socio-economic classes were underrepresented.^25^ Risk factors such as age and BMI may be distributed differently in low-income pregnant women. This may have introduced a bias, most probably towards a lower prevalence of incontinence than in the total target population. There is, however, no reason to believe that there was a selection on the basis of incontinence status since the MoBa was a survey covering many topics, and urinary incontinence questions only being a minor issue. We believe that the effect estimates for risk factors investigated in this study were not affected by a significant selection bias. A strength of the MoBa study is that the participating women remained in the study; of women responding on questionnaire in week 30 of pregnancy as many as 87% completed the questionnaire 6 months postpartum.^25^

To inform clinicians, we present detailed data for non-elective CS by splitting this group into three (those who were intended to deliver vaginally, those who were intended to deliver by elective CS and an unspecified group). There were, however, no significant differences between these groups. When interpreting these data, one has to take into account that the latter two groups contained small numbers of participants. Several studies support our findings in that the birthweight,^10^,^13^,^30^ head circumference,^9^,^13^ sex,^11^ Apgar score,^12^ prolonged labour,^9^,^30^ induction of labour,^32^ fetal presentation at delivery^10^–^12^ and perineal tear grade 3–4^10^,^13^,^32^ are weak or not at all risk factors for urinary incontinence, and these factors did not confound the results in the present study. The Medical Birth Registry obtains information on mode of delivery. We have no information regarding indications for non-elective CS; hence, some confounding by indication may be the case. No further information was obtained on which instrumental delivery failed and resulted in non-elective CS or at what stage of delivery non-elective CS was carried out. This kind of missing information is a limitation of this study. The proportion of CS (14.3%) and forceps (2.4%) in this study were quite similar to the proportions for all deliveries in Norway as a whole during this time period (CS 13.5–16.5% and forceps 1.3–1.9%). In adjusted analyses, the association between delivery mode and urinary incontinence postpartum was stronger compared with unadjusted analyses, probably reflecting higher mean BMI and age among women having CS.

We found significant differences in prevalence of urinary incontinence depending on continence status in pregnancy and mode of delivery. However, after adjustment and transferring the estimates to RRs rather than odds ratios, the differences were minor. Odds ratio is a misleading outcome measure in studies with high prevalence in the unexposed group, like in this study.^29^ We recommend the procedure of transferring odds ratio to RR for future studies on groups with high prevalence of incontinence. Also, one should be careful to interpret the results into a clinical setting, as this is a study comprising women carefully selected as being primigravid and continent before pregnancy.

We used a symptom-based questionnaire based on definitions of the ICS.^27^ Although the questionnaire was not validated *per se*, the questions were similar to those of validated instruments.^37^

A major strength in this very large observational cohort is the narrow confidence intervals indicating high precision of the results. The nulliparous continent pelvis represents the best available clinical model of the unexposed pelvis,^26^ and our design is thereby the best to assess the risk of urinary incontinence associated with pregnancy and delivery.

Elective CS was associated with less risk of urinary incontinence postpartum compared with SVD. Women who were continent during pregnancy had statistically significant lower prevalence of urinary incontinence postpartum compared with those who were incontinent. There were, however, no statistically significant differences in risks between women who were continent and incontinent in pregnancy depending on mode of delivery. In conclusion, our findings indicate that the association between mode of delivery and continence status postpartum was not influenced by incontinence status in pregnancy. Prediction of a group with high risk of urinary incontinence according to mode of delivery cannot be based on continence status in pregnancy.

### Disclosure of interest

None.

### Contribution to authorship

S.L.W.: performed analyses and did the main interpretation of data. He wrote the article and approved the final version. G.R.: came up with the research question, helped considerably with the analysis, did the major supervising and revising of the article and have given final approval of the version being published. K.B.: had access to the data set. She has given critical feedback on the article along the way. She has given her final approval of the version being published. S.H.: expressed the objective of the study and the methods to be used, helped with interpretation of the results. He critically revised and commented on drafts of the manuscript. He has given final approval of the version being published.

### Details of ethics approval

The Norwegian Data Inspectorate approved the MoBa study in 1996 and renewed the approval in 2003. The Regional Ethics Committee for Medical Research, Health Region II, Norway, has also endorsed the project. Date of approval: 10 September 1998. Reference number S-95113. Our subanalysis in this project itself does not need its own approval. The Regional Ethics Committee for Medical Research has stated: ‘We hereby confirm that the project “Urinary incontinence in women during pregnancy and after delivery. The Norwegian mother and child cohort study” by project supervisor S.L.W., at the Section for General Practice, Department of Public Health and Primary Health Care, University of Bergen, Norway, is exempted from review by the Regional Committee for Medical and Health Research Ethics, Western-Norway’.

### Funding

The article is supported financially by Western Norway Regional Health Authority.
